# Coagulase-positive methicillin-resistant *Staphylococcus aureus* circulating in clinical mastitic goats in Bangladesh

**DOI:** 10.14202/vetworld.2020.1303-1310

**Published:** 2020-07-11

**Authors:** Eaftekhar Ahmed Rana, Tridip Das, Avijit Dutta, Mizanur Rahman, Mohammad Bayazid Bostami, Nasima Akter, Himel Barua

**Affiliations:** 1 Department of Microbiology and Veterinary Public Health, Chattogram Veterinary and Animal Sciences University, Chattogram, Bangladesh; 2 Teaching and Training Pet Hospital and Research Center, Chattogram Veterinary and Animal Sciences University, Chattogram, Bangladesh; 3 Department of Dairy and Poultry Science, Chattogram Veterinary and Animal Sciences University, Chattogram, Bangladesh

**Keywords:** Antimicrobial resistance, clinical mastitis, coagulase-positive *Staphylococcus aureus*, goat, methicillin-resistant *Staphylococcus aureus*

## Abstract

**Background and Aim:**

*Staphylococcus aureus* is argued as one of the principal organisms responsible for mammary gland infection in lactating goats, causing both clinical and subclinical mastitis. Being highly zoonotic potential, pathogen emergence of methicillin-resistant *S. aureus* (MRSA) has a significant clinical impact on treatment and management of clinical mastitis. We conducted a cross-sectional study to investigate the prevalence of coagulase-positive *S. aureus* (CoPS), antimicrobial resistance profile of *Staphylococcus* spp., prevalence of MRSA, and association between different clinical parameters with CoPS.

**Materials and Methods:**

A total of 67 clinical mastitic goats were sampled based on clinical examination and California mastitis test. Standard bacteriological methods were performed to isolate and identify *Staphylococcus* spp. CoPS were confirmed by *nuc* gene using polymerase chain reaction (PCR). All staphylococcal isolates were further examined for antimicrobial susceptibility testing by disk diffusion method. MRSA was confirmed based on *mecA* gene-based PCR.

**Results:**

Here, 49 (73.13%; 95% confidence interval [CI], 61.41-82.35) samples were positive for *Staphylococcus* spp., of which 17 (34.69%; 95% CI, 22.88-48.73) isolates were CoPS and rest of the isolates (32; 65.30%; 95% CI, 51.27-77.12) were identified as coagulase-negative *Staphylococcus* spp. (coagulase-negative staphylococci [CNS]). Both, CoPS and CNS isolates displayed the highest resistance against tetracycline (76.47% and 75%, respectively) and oxacillin (70.58% and 68.75%, respectively). Notably, all staphylococcal isolates were multidrug-resistant (showed resistance to ≥3 classes of antimicrobials). *mecA* gene was found in 6 (8.96%; 95% CI, 3.84-18.52) CoPS isolates indicating MRSA strains. Among different clinical parameters, presence of high body temperature (p<0.05), firm udder consistency (p<0.01), bloodstained milk (p<0.00), and pus in milk (p<0.00) were significantly associated with the presence of CoPS in mastitic caprine milk.

**Conclusion:**

To the best of our knowledge, this is the first report of MRSA isolated from clinical caprine mastitis cases in Bangladesh. The findings of this study would help in cautious selection as well as administration of antimicrobials for therapeutic management of mastitic goats.

## Introduction

Mastitis is one of the most challenging production diseases for lactating animals, particularly for bovine, ovine, and caprine all over the world. Diverse groups of pathogens, especially bacteria, frequently act as etiological agent for mammary gland infections. Among the pathogens, *Staphylococcus aureus* is the most frequently isolated contagious bacterial pathogen [1]. Due to carriage of multiple virulence factors, of *S. aureus* is responsible for acute, chronic, and gangrenous mastitis in lactating goats [2]. In this regard, for clinical recovery, caprine mastitis is treated with different groups of broad-spectrum antimicrobials that create potential force to kill or inhibit the growth of bacteria. Due to continuous exposure of antimicrobial agents, it ultimately creates selection pressure and results in emergence of resistant strain of the bacteria [3].

In recent years, methicillin-/oxacillin-resistant staphylococcal strains create a serious clinical perplexity to treat the caprine mastitis. Methicillin-resistant *S. aureus* (MRSA) strains possess *mecA* gene, which codes for the expression of penicillin-binding protein (PBP2a, an altered form of PBP2), resulting decreased affinity of methicillin as well as all β-lactam antibiotics to bind with bacterial cell wall [4]. Ultimately, *S. aureus* has developed multidrug resistance with a wide variation from animal to animal [5] and makes difficulties in clinical management of mastitis. As a result, multidrug-resistant (MDR) mastitis pathogens, particularly methicillin-resistant staphylococcal strains, are considered a well-documented threat to the dairy goat production [6]. Although livestock-associated MRSA (LA-MRSA) was reported in very early period in farm animals [7], reports on MRSA have tremendously been increased day by day across the globe [8]. The emergence of MRSA in food animals is alarming for animal professionals, farmers, as well as persons who come in close contact with animals regularly [6,9]. On the other hand, animals harboring MRSA continuously disseminate this MDR potential zoonotic pathogen toward the human and other animal communities. Furthermore, series of reports mention that both pet and food animals act as potential reservoir of human infections with MRSA [10-13]. From public health aspect, there is a possible chance of transmission of MRSA strains from animals to humans who rear animals or consume the ultimate farm products [8,9,13,14]. However, the occurrence of MRSA as a causal agent of caprine mastitis remains underreported, if not unreported in Bangladesh. Hence, it is a timely demand to investigate the presence of MRSA in mastitis affected lactating goats aiming to formulate effective preventive measures guideline.

The study was designed to investigate the prevalence of coagulase-positive *S. aureus* (CoPS) as well as antimicrobial sensitivity profile of *Staphylococcus* spp. with molecular identification of MRSA and, finally, to find out the association between CoPS and different clinical parameters of mastitic goats.

## Materials and Methods

### Ethical approval and Informed consent

In this study, we used only mastitic milk that was collected with minimum discomfort of animals for clinical inspection of mastitic milk for diagnostic as well as treatment purposes in Teaching Veterinary Hospital (TVH). Therefore, verbal permission from animal owners and hospital director of Chattogram Veterinary and Animal Sciences University (CVASU) was taken to conduct the research.

### Study area and duration

A cross-sectional study was conducted at TVH-CVASU, Chattogram, Bangladesh, for a period of 10 months (July 2018-April 2019). Goats from different areas of Chattogram are regularly brought to TVH-CVASU for different therapeutic purposes. The targeted population was the mastitic goats among them.

### Data collection

A pre-tested questionnaire was used to collect the mastitic goat’s data from the respective owner. Clinical data for each case were recorded during clinical examination of the patients (mastitic goat) at the hospital.

### Assessment of clinical mastitis

Clinical mastitis of goat was primarily diagnosed by clinical examination, including palpation and observation of the udder. Visual confirmation of clinical mastitis was based on the presence of indurations of one or both quarters, swelling of udder, abnormal secretion of milk including flaky clots, discoloration of milk including decreased surface tension, and blood stained secretion from udder. Presence of redness, heat, and pain found on palpation of udder were treated as clinical mastitis. In addition, goats with high body temperature, loss of appetite, decrease milk production, dullness, and depression were considered as associated clinical signs of clinical mastitis.

California mastitis test (CMT) was performed for further confirmation of clinical mastitis cases. CMT was performed according to the manufacturer’s instructions (Leukocytest^®^, Synbiotics Corporation, France). Approximately 2 ml of milk was taken from individual teat of the goat in each well of the test plate. Then, equal amount (2 ml) of CMT reagent was added in the test well and circular motion was made for mixing the reagents and milk. Finally, the test results were interpreted as negative (somatic cell count [SCC] score of ≤100,000 cells/ml), weak positive (1+; SCC score of >100,000-500,000 cells/ml), distinct positive (2+; SCC score of >500,000-1,000,000 cells/ml), and strong positive (3+; SCC score of ≥1,000,000 cells/ml) according to the manufacturer’s instruction [15].

### Bacterial isolation and identification

For bacterial isolation, first, 20 μl CMT-positive mastitic milk sample was cultured on 5% bovine blood agar (Oxoid Ltd., UK). The inoculated blood agar plates were incubated at 37°C for 48 h aerobically. Colonies displaying the characteristic growth of staphylococci on blood agar (pigmented, raised, medium-sized, and hemolytic) were primarily selected for phenotypic characterization. The presumptive staphylococci colonies were subcultured onto mannitol salt agar (Oxoid Ltd., UK) and incubated for 24 h at 37°C. Presumptive colonies were further confirmed using Gram staining and catalase test. All staphylococcal isolates were subjected to the tube coagulase test, as described by Carfora *et al*. [16]. Isolates were then stored at −80°C using 50% glycerol until further examination.

### Molecular identification of S. aureus by nuc gene polymerase chain reaction (PCR)

All staphylococcal isolates were subcultured on blood agar. Bacterial genomic DNA was extracted using crude boiling lysis method [17]. Finally, CoPS was confirmed by the PCR amplification of *nuc* gene (a characteristic thermonuclease gene of *S. aureus*). For PCR, the primer sequences used were au-F3 (Forward) 5̕ TCGCTTGCTATGATTGTGG 3̕ and au-R (Reverse) 5̕ GCCAATGTTCTACCATAGC 3̕. The amplification condition was as follows: Initial denaturation at 95°C for 2 min, followed by 30 cycles of final denaturation at 95°C for 30 s, annealing at 56°C for 35 s, initial extension at 72°C for 60 s, and final extension at 72°C for 2 min [18]. For positive and negative controls, *S. aureus* ATCC 29213 strain and nuclease-free water were used, respectively.

### Antimicrobial susceptibility testing

All staphylococcal isolates were tested against 13 different antimicrobials of various groups using disk diffusion method [19]. The antimicrobials used were ampicillin (10 μg), ciprofloxacin (5 μg), gentamicin (30 μg), tetracycline (30 μg), erythromycin (15 μg), oxacillin (5 μg), amoxicillin (10 μg), cefaclor (30 μg), sulfamethoxazole- trimethoprim (23.75+1.25 μg), cefoxitin (10 μg), ceftriaxone (10 μg), penicillin (10 IU), and streptomycin (100 μg) (Oxoid, Basingstoke, UK). For each isolate, the zone of inhibition was measured and interpreted as susceptible (S), intermediate (I), and resistant (R) according to CLSI guidelines [19]. Isolates resistant to ≥3 classes of antimicrobials were considered as MDR [5].

### Molecular identification of mecA gene

*S. aureus* isolates showing resistance to oxacillin and cefoxitin were primarily considered as MRSA [20]. Only phenotypically resistant isolates were further investigated for the presence of *mecA* gene by PCR, as described by Larsen *et al*. [21]. Primers used were *mecA* P4 (Forward) 5̕ TCCAGATTACAACTTCACCAGG 3̕ and *mecA* P7 (Reverse) 5̕ CCACTTCATATCTTGTAACG 3̕. The PCR amplification program consisted of initial denaturation at 94°C for 15 min, followed by 35 amplification cycles at 94°C for 30 s, annealing at 55°C for 45 s, and final extension at 72°C for 1 min [21]. MRSA ATCC 33591 strain and nuclease-free water were used as positive and negative controls, respectively.

### Statistical analyses

Data obtained from both bacteriology and questionnaires were entered into Microsoft Excel 2007 and analysis was performed using “R” Program (version 3.5.1) [22]. The prevalence was calculated, dividing the number of positive cases by the total number of goats sampled. All recorded clinical parameters were analyzed for one target outcome of *nuc* gene-positive *S. aureus* (CoPS). First, univariable analysis was performed to identify possible clinical condition(s) associated with *nuc* gene-positive *S. aureus*. Any clinical parameter(s) having p≤0.20 was entered into multivariable logistic regression model. Forward stepwise selection approach was used to build the final model. Variables with p≤0.05 were considered as significant and kept in the final model. The logistic regression analysis was performed using the glmer function from the lme4 package in R version 3.5.1 [22].

## Results

### Categories of clinical mastitis based on CMT

Out of 67 clinical cases, 48 (71.64%; 95% CI, 59.85-81.09) cases were identified as distinct positive (2+), whereas only 11 (16.41%; 95% CI, 9.25-27.23) and 8 (11.94%; 95% CI, 5.92-22.09) cases were diagnosed as weak positive (1+) and strong positive (3+), respectively. However, none of the clinical cases were found CMT negative.

### Bacteriological analyses

**Figure-1:**
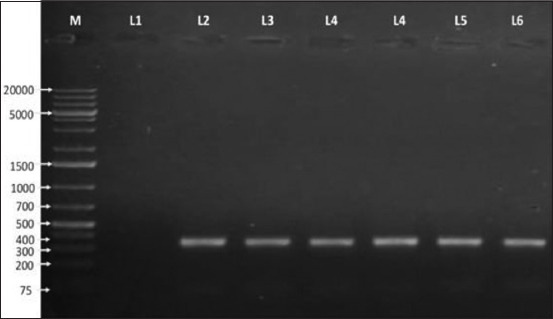
Agarose gel electrophoresis (1% agarose) image of polymerase chain reaction products of coagulase-positive *Staphylococcus aureus* (CoPS) isolates showing specific amplified bands 359 bp. M=1 kb plus DNA marker, L1=Negative control, L2=Positive control (ATCC 29213), L3-L6=CoPS isolates.

Overall, 49 (73.13%, 95% CI, 61.41-82.35) samples were found to be positive for staphylococci, of which 17 (34.69%, 95% CI, 22.88-48.73) isolates carried *nuc* gene (Figure-1) and thus identified as CoPS, and remaining 32 (65.30%, 95% CI, 51.27-77.12) isolates were coagulase-negative staphylococci (CNS) (Table-1).

**Table-1:** Frequency of CoPS and CNS with their antimicrobial sensitivity profile.

*Staphylococcus* isolates	Frequency (%)	Antimicrobial susceptibility	CFO	CEF	AMP	ERY	SUL	CIP	OXA	TET	AMC	CFC	GEN	PEN	STP
CoPS	17 (34.69)	Sensitive (%)	58.82	70.58	29.41	41.17	52.94	58.82	29.41	23.52	41.17	52.94	35.29	47.05	41.18
		Resistant (%)	41.17	29.41	70.58	58.82	47.05	41.17	70.58	76.47	58.82	47.05	64.7	52.94	58.82
CNS	32 (65.31)	Sensitive (%)	65.63	43.75	40.62	28.12	59.37	65.63	31.25	21.87	62.5	50	43.75	40.62	43.37
		Resistant (%)	34.37	56.25	59.37	71.87	40.62	34.37	68.75	78.13	37.5	50	56.25	59.37	56.63

CoPS=Coagulase-positive * Staphylococcus aureus*, CNS=Coagulase-negative staphylococci, where, CFO=Cefoxitin, CEF=Ceftriaxone, AMP=Ampicillin, ERY=Erythromycin, SUL=Sulfamethoxazole-trimethoprim, CIP=Ciprofloxacin, OXA=Oxacillin, TET=Tetracycline, AMC=Amoxicillin, CFC=Cefaclor, GEN=Gentamicin, PEN=Penicillin, STP=Streptomycin

### Antimicrobial susceptibility profiles

**Figure-2:**
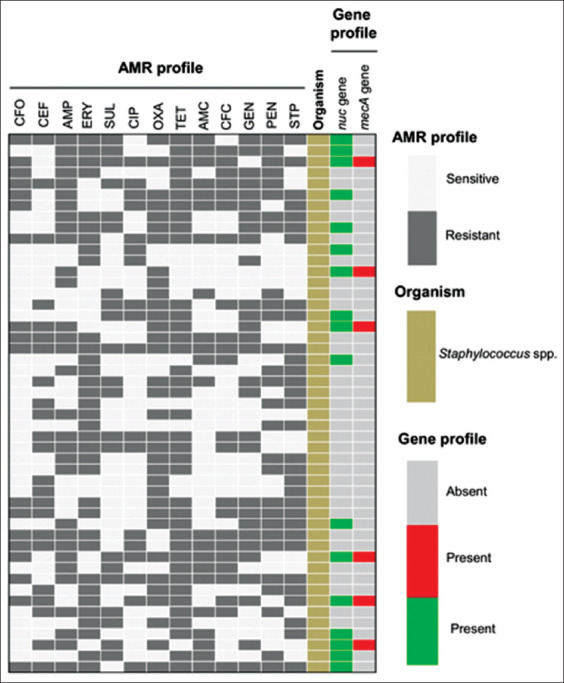
Heat map showing antimicrobial resistance phenotype, coagulase-positive gene (*nuc*), and resistance gene (*mecA*) profile of *Staphylococcus* isolates (n=49) from different clinically mastitis affected goats. All intermediate resistant isolates are considered as susceptible.

All the CoPS and CNS isolates were found to be MDR (i.e., resistant to ≥3 antimicrobial classes) (Figure-2). In both cases, the highest resistance was observed against tetracycline (76.47%, 95% CI, 52.23-90.95 and 78.13%, 95% CI, 60.96-89.27, respectively) followed by oxacillin (70.58%, 95% CI, 46.57-87.01 and 68.75%, 95% CI, 51.32-82.16, respectively). More than 58% isolates were resistant to gentamycin, erythromycin, amoxicillin, and streptomycin in case of CoPS isolates (Table-1). Whereas, around 60% CNS isolates displayed resistance against ampicillin, penicillin, gentamycin, and streptomycin (Table-1). However, 70.58% CoPS isolates were susceptible to ceftriaxone and more than 65% CNS isolates were susceptible to cefoxitin and ciprofloxacin, respectively (Table-1).

### MRSA

**Figure-3:**
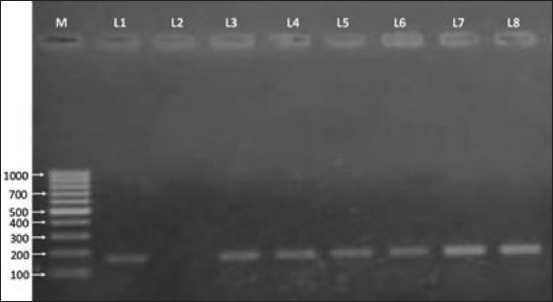
Gel electrophoresis image of polymerase chain reaction products of methicillin-resistant *Staphylococcus aureus* (MRSA) isolates showing specific amplified bands 162 bp on 1% agarose gel. M=100 bp DNA marker. L3-L8=MRSA-positive isolates, L2=Negative control, L1=Positive control (ATCC 33591).

Of the 67 clinical mastitic goats, 6 (8.96%, 95% CI 3.84-18.52) were positive for *mecA* gene. Notably, all *mecA* genes were carried by CoPS and classified as MRSA (Figure-3).

### Association of CoPS with different clinical parameters of mastitic goats

The univariable analysis identified nine clinical parameters (p≤0.2) to be associated with the carriage of *nuc* gene-positive *S. aureus* in mastitic goats (Table-2). Of them, high body temperature (p<0.05), firm udder consistency (p<0.01), bloodstained milk color (p<0.00), and presence of pus in milk (p<0.00) were statistically significant clinical conditions associated with the presence of CoPS. In the subsequent multivariable analysis, only one clinical sign (bloodstained milk color) was significantly associated (p<0.00) with the carriage of CoPS.

**Table-2:** Association of *nuc* gene carriage *S. aureus* positive clinical mastitis with different clinical parameters of mastitis affected goats.

Variables	Covariables	No. of mastitic goats	No. of *S. aureus* positive for *nuc* gene (%)	95% CI	p-value
Body temperature	Higher (≥104°F)	34	11 (32.35)	19.04-49.25	0.05^*^
	Optimum (101°-103°F)	27	3 (11.12)	3.03-28.88	
	Subnormal (≤100°F)	6	3 (50)	18.76-81.24	
Udder consistency	Firm	31	6 (19.35)	8.82-36.65	0.01^*^
	Normal	24	4 (16.67)	6.07-36.47	
	Soft	12	7 (58.34)	31.89-80.74	
Udder color	Blackish	6	3 (50)	18.76-81.24	0.17
	Normal	35	10 (28.57)	16.19-45.20	
	Reddish	26	4 (15.38)	5.53-34.15	
Udder inflammation	Yes	44	11 (25)	14.43-39.59	0.92
	No	23	6(26.08)	12.26-46.76	
Pain in udder	Yes	49	13 (26.53)	16.10-40.37	0.71
	No	18	4 (22.23)	8.47-45.75	
Milk color	Bloodstained	23	14 (60.86)	40.73-77.90	0.00^*^
	Normal	10	1(10.0)	0.01-42.60	
	Watery	34	2 (5.88)	0.65-20.07	
Udder temperature	Cold	4	2 (50)	15.0-85.0	0.49
	Hot	45	11 (24.45)	14.08-38.82	
	Normal	18	4 (22.23)	8.47-45.75	
Flack in milk	Yes	49	14 (28.57)	17.76-42.50	0.32
	No	18	3 (16.67)	5.01-40.05	
Pus in milk	Yes	35	14 (40.0)	25.52-56.46	0.00^*^
	No	32	3 (9.37)	2.46-25.0	
Feed intake	Optimum	20	3 (15.0)	4.39-36.88	0.21
	Decrease	41	11 (26.82)	15.56-42.07	
	Anorexia	6	3 (50.0)	18.76-81.24	
Heart rate	Normal	28	5 (17.85)	7.41-36.06	0.11
	Increase	34	12 (35.29)	21.42-52.15	
	Decrease	5	0 (0.00)	0-48.91	
Respiration rate	Normal	31	6 (19.35)	8.82-36.65	0.13
	Increase	31	11 (35. 48)	21.53-53.12	
	Decrease	5	0 (0.00)	0-48.91	
Milk production	Optimum	12	1 (8.33)	0.01-37.53	0.13
	Decrease	55	16 (29.09)	18.70-42.21	
Appearance	Healthy	39	9 (23.07)	12.44-38.54	0.61
	Dull and depressed	28	8 (28.57)	15.07-47.24	
Gangrene in udder	Absence	60	14 (23.34)	14.32-35.55	0.26
	Presence	7	3 (42.85)	15.75-75.02	

CI=Confidence interval, significance *(≤0.05)

## Discussion

*S. aureus* is the most significant and commonly isolated mastitis-causing organism globally, including Bangladesh. In this study, bacteriological examination revealed that overall prevalence of staphylococci in caprine mastitis cases was 73.13% and it showed close agreement with the previous findings of 73.73% by Amin *et al*. [23] and 51% by Vyletelova *et al*. [24]. On the contrary, comparatively lower prevalence is also reported by Danmallam and Pimenov (20%), Sarker and Samad (38.98%), and Pirzada *et al*. (36.84%) [25-27]. As *Staphylococcus* spp. is predominant commensal pathogen in goat bodies, for this reasons, it might be frequently associated with clinical and subclinical mastitis of goat. At the same time, healthy mammary gland environment may also favor the growth of this organism and presence of virulence factors in staphylococci subsequently aggravates the condition. The prevalence of CoPS was found 34.69%, which is closely similar to the previous studies of Vyletelova *et al*. [24] and Haftay *et al*. [28] where they reported found 32.2% and 23.4% prevalence of CoPS in clinical caprine mastitis cases, respectively. In bacteriological studies, 65.30% isolates were found CNS, which is in accordance with the previous reports [27,29]. According to Bochev and Russenova [30], about 80.2% CNS are associated with subclinical mastitis in goat. The variation of prevalence might be due to geographical location, previous carriage of subclinical mastitis, variation in hygienic practices, stage of lactation, number of parity, etc. However, the methods followed by the different researchers could also be a possible reason for the variation in the prevalence of the pathogens.

A significant increase of SCC with the presence of staphylococci is one of the important features of both clinical and subclinical mastitis along with the presence of different mastitis associated clinical signs [31]. About 71.64% mastitis cases were ranked as distinct positive (2+) and SCC of the clinical samples was >500,000-1,000,000 cells/ml using CMT, which was supported by previous findings [32,33]. In general, *S. aureus* is mainly responsible for clinical, subclinical, and chronic mastitis, often characterized by a marked increase in SCC [1].

Among the 13 antimicrobials tested against both CoPS and CNS, tetracycline was the least effective therapeutic option. However, comparatively much lower resistance profile of goat CoPS and CNS isolates to tetracycline is described by Bochev and Russenova and Virdis *et al*. [30,34]. They observed resistance rates of 16% and 24%, respectively. The penicillin and ampicillin also showed marked resistance against *S. aureus* that was agreed to results reported by Bochev and Russenova [30] and Virdis *et al*. [34] who found 75% and 36% resistance, respectively. The resistance to penicillin and ampicillin found in this study might be of great concern since these drugs represent the main antibiotic group which is recommended for staphylococcal mastitis treatment. However, representative drug(s) from other groups of antimicrobials such as erythromycin (macrolides), gentamycin and streptomycin (aminoglycosides), and sulfamethoxazole-trimethoprim (sulfonamides) showed remarkable resistance against staphylococcal isolates tested in this study.

Our study first reports the prevalence and circulation of MRSA in mastitic goats in Bangladesh. Since the study was conducted in veterinary hospital where only clinical goats get admitted, we found a very low MRSA prevalence, with only 6 (8.96%) out of 67 cases tested. However, 20% MRSA prevalence in mastitic goat was previously described by Bochev and Russenova [30]. In Italy, Carfora *et al*. [35] and Cortimiglia *et al*. [36] reported a very low MRSA prevalence in sheep (0.34%) and goat (1.3%) farms, respectively. Whereas, MRSA prevalence rate varying from 0 to 29% was reported earlier by Vanderhaeghen *et al.*, Schlotter *et al.*, and Luini *et al*. [37-39] in mastitis affected dairy cattle herds.

In this study, carriage of staphylococci and clinical examination of the mastitic udder was carried out. Through clinical examination of goat udder, we identified different clinical parameters which commonly denote mastitis. In goat, CoPS is mainly associated with clinically evident mastitis and it is related to different clinical findings such as high body temperature, firm udder consistency, bloodstained milk, and presence of pus in mastitic milk [1,40,41]. Hence, clinical examination of mastitic patient is the first tool of diagnosis which is easy, economical, and rapid as well. In our opinion, clinical examination of the goat udder is an utmost important diagnostic tool along with the classical microbiological approach of culturing suspected milk sample to diagnose clinical mastitis.

However, potential transmission of MRSA from animal to human has already been published, indicating a serious risk might be involved with this zoonotic pathogen [8]. Transmission of MRSA from goat to farmers, veterinary professionals, as well as milk processing personnel will result in serious public health consequences in the end.

## Conclusion

In the present study, the prevalence of CoPS and CNS found in mastitic goat was 34.69% and 65.30%, respectively. Between CoPS and CNS, CNS was the most frequent bacterial group associated with clinical mastitis. An exigent issue is that both the CoPS and CNS were MDR. CoPS were carrying *mecA* gene (8.96%) which has potential zoonotic significance. The significant clinical conditions associated with the presence of CoPS were increased body temperature, firm udder consistency, bloodstained milk, and pus in milk that help in definite diagnosis and treatment of mastitic goat. Hence, it can be said that the identification of the specific organism(s) responsible for the caprine clinical mastitis and exploration of antimicrobials resistance profile of these organisms are of utmost importance for the development of an effective control program and treatment strategies.

## Authors’ Contributions

EAR Planned, designed, and organized the present study, conducted the laboratory task, including isolation and identification of bacteria, molecular method (PCR), statistical analyses, and primary drafting of the manuscript. TD, AD, and NA were responsible for the literature review, results interpretation, and guided for the manuscript preparation. MR and MBB were responsible for the sample and data collection as well as clinical examination of the animals. HB Supervised the full study and revised the final manuscript. All authors have read and approved the final manuscript.
